# Case Report: Giant lung hamartoma : An usual cause of lobectomy in a five-year child

**DOI:** 10.12688/f1000research.146993.2

**Published:** 2024-09-24

**Authors:** Sabrine Louhaichi, Besma Hamdi, Imen Bouacida, Yessmine Haddar, Sarra Trimech, Jamel Ammar, Aida Ayadi, Agnès Hamzaoui, Baccouche Ines, Adel Marghli

**Affiliations:** 1 Department of Respiratory Diseases B. Abderrahmen Mami Hospital, Ariana, Tunisia; 2 Faculty of Medicine of Tunis, Laboratory Research 19SP02; Chronic Pathologies: From Genome to Management Tunis El Manar University, Tunis, Tunisia; 3 Department of Thoracic and Cardiovascular Surgery Abderrahmen Mami Hospital, Ariana, Tunisia; 4 Department of Pathology, Department of Pathology, Abderrahmen Mami Hospital, Ariana, Tunisia; 5 Department of Radiology, Abderrahmen Mami Hospital, Ariana, Tunisia

**Keywords:** lung tumor, case report, children, surgical intervention, hamartoma

## Abstract

Pulmonary hamartomas are the most common benign tumors of the lung in adults. They are usually asymptomatic because of their small size and their slow-growing character. We report the case of a 5-year-old child presenting with a giant lung mass causing recurrent right pneumonia. Surgical resection with middle lobectomy was performed. Final histology revealed pulmonary hamartoma with predominant adenofibromatous and lipomatous differentiation.

## Introduction

Lung hamartomas are benign pulmonary tumors characterized by an incidental finding in most cases. ^1^ Compocased of a mixture of variant mesenchymal elements, it is more frequently seen in male adults. Pediatric cases are extremely rare. ^2^^,^^3^ Herein we report the case of a pulmonary hamartoma revealed by persistent pneumonia in a five-year-old child.

## Case report

**Figure 1.**
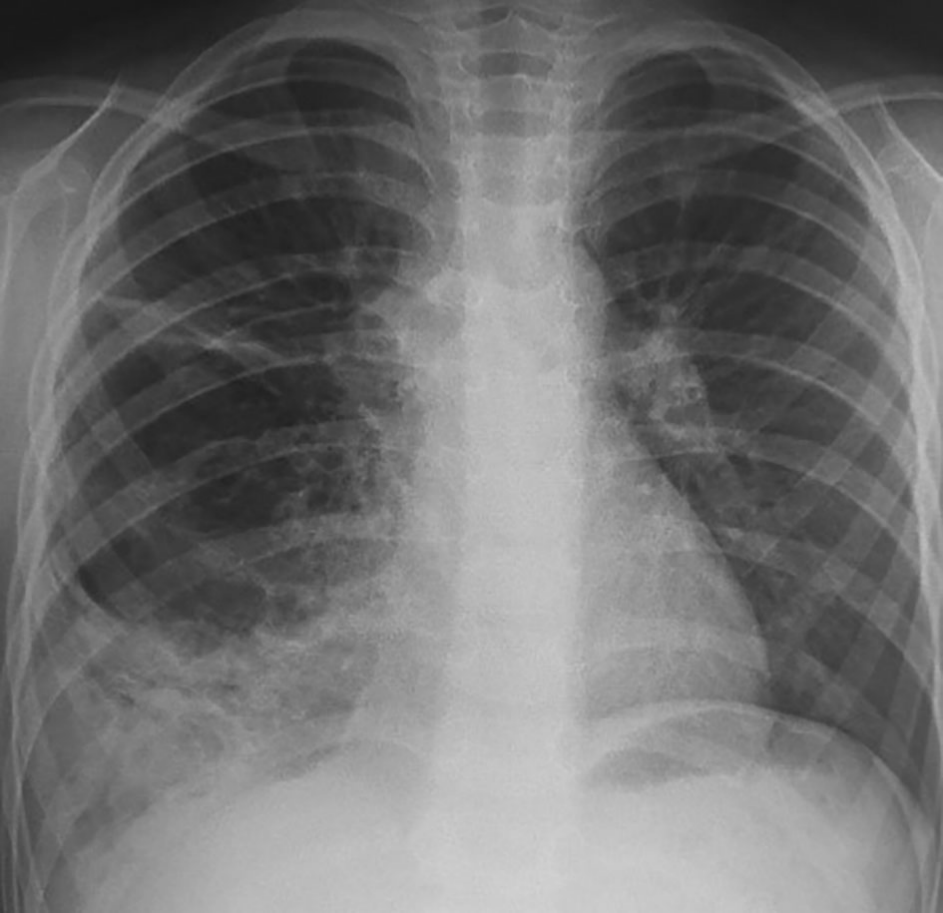
Chest radiograph view demonstrating a heterogeneous opacity in the middle and lower zones of the right lung.

A five-year-old child was referred to our department in September 2023 because an abnormal pulmonary density of the lower right hemithorax. His past medical history revealed recurrent admissions for right pneumonia during the last two years. The patient complained of chronic productive cough without chest pain or hemoptysis. Physical examination did not reveal abnormalities apart from a decrease in breath sounds in the right lung. Chest radiography revealed a heterogeneous right opacity above the diaphragm as showed in Figure 1.

**Figure 2.**
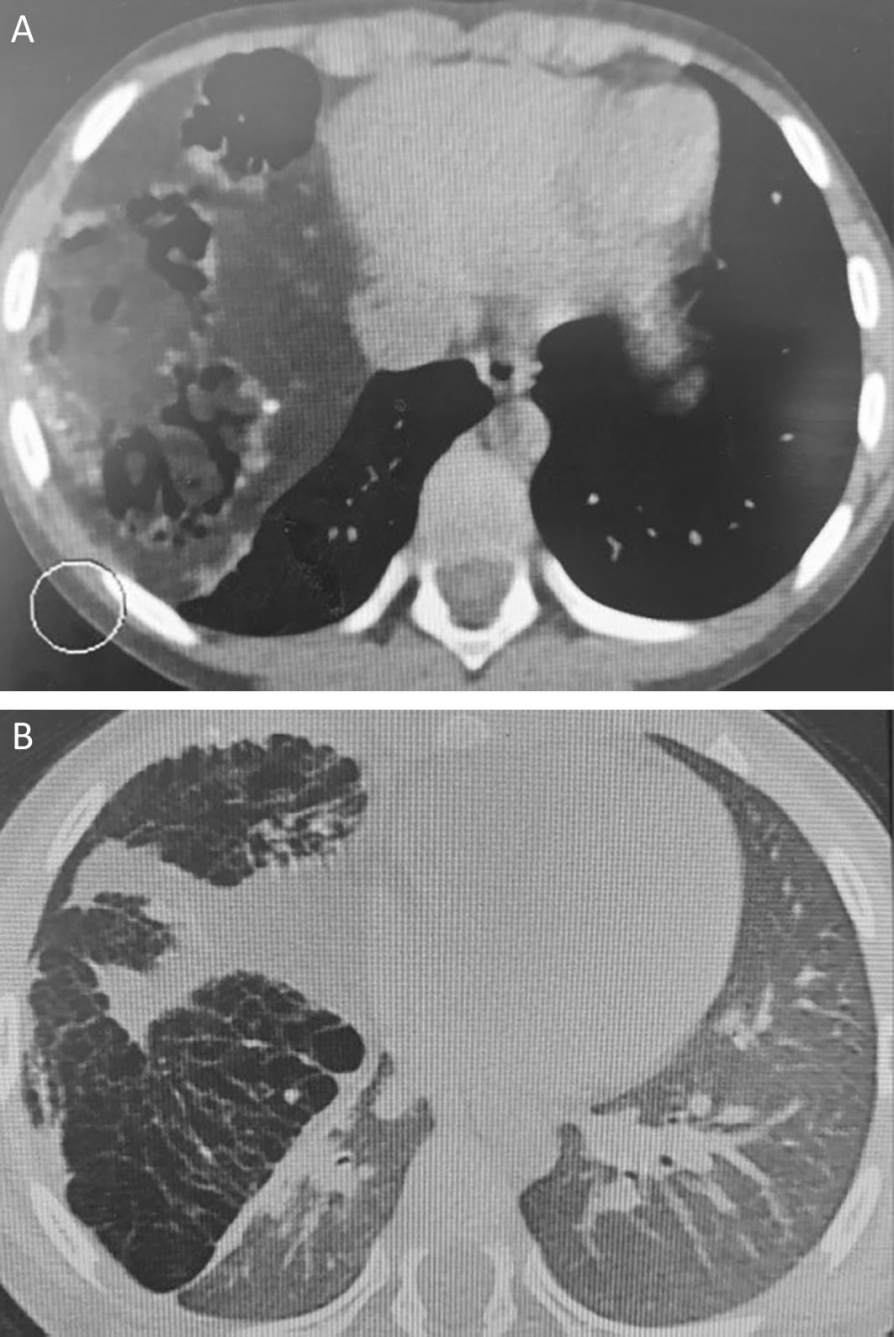
Pulmonary hamartoma: a contrast-enhanced lung CT scan is showing a large, lobulated soft-tissue density mass with foci of low attenuation and calcification with compression of the right lower lobe in mediastinal window (A) and lung window (B).

Chest computed tomography revealed a giant cystic and solid mass measuring 122 × 80 × 102 mm compressing the right middle and lower lobes. This mass contained tissular, fatty, and calcified elements, along with multiple airy cysts, suggesting a giant pulmonary hamartoma ( Figure 2).

**Figure 3.**
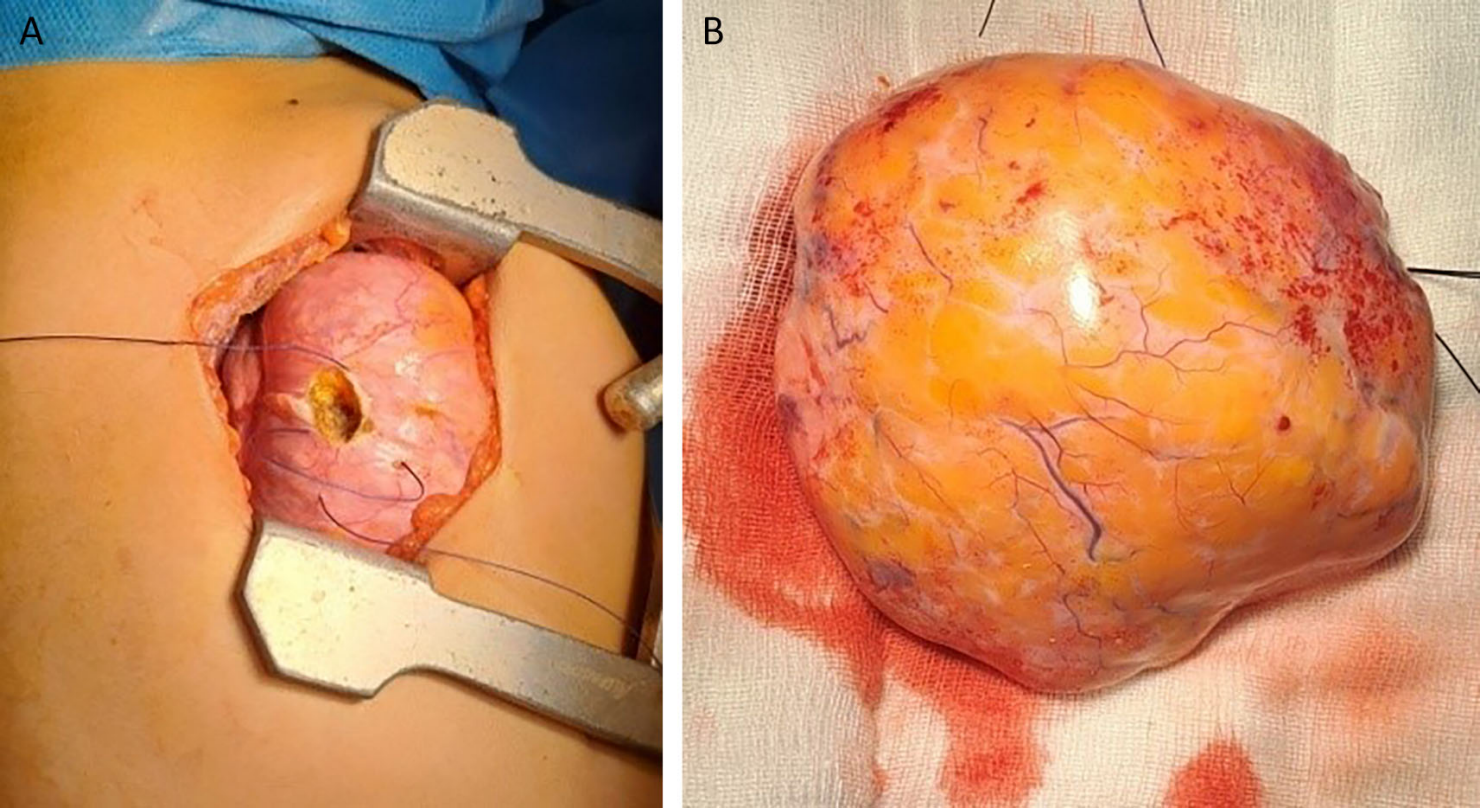
Resection of the tumor (A). The hamartoma after resection (B).

Therapeutic options were discussed in a multidisciplinary reunion and surgery was decided. It was performed under general anaesthesia. The patient underwent a right lateral thoracotomy. During exploration, the mass occupied two-thirds of the right thorax and compressed the upper and lower lobes ( Figure 3) as well as the mediastinum. It was carefully mobilized. It doesn’t invade the mediastinum and the phrenic nerve was identified and preserved. The fatty mass depended on the middle lobe, which was a small strip of destroyed lung parenchyma. The surgical strategy was, to begin with an atypical resection removing the bloc of the mass followed by a complete right middle lobectomy. The dissection of the middle lobe arteries was challenging due to the destroyed tissue. The anatomical resection was successfully achieved and the patient was extubated immediately in the operating room. There were no anaesthetic complications during the procedure. The postoperative course was uneventful. The chest drain tube was removed three days postoperatively and the patient was discharged our days later.

**Figure 4.**
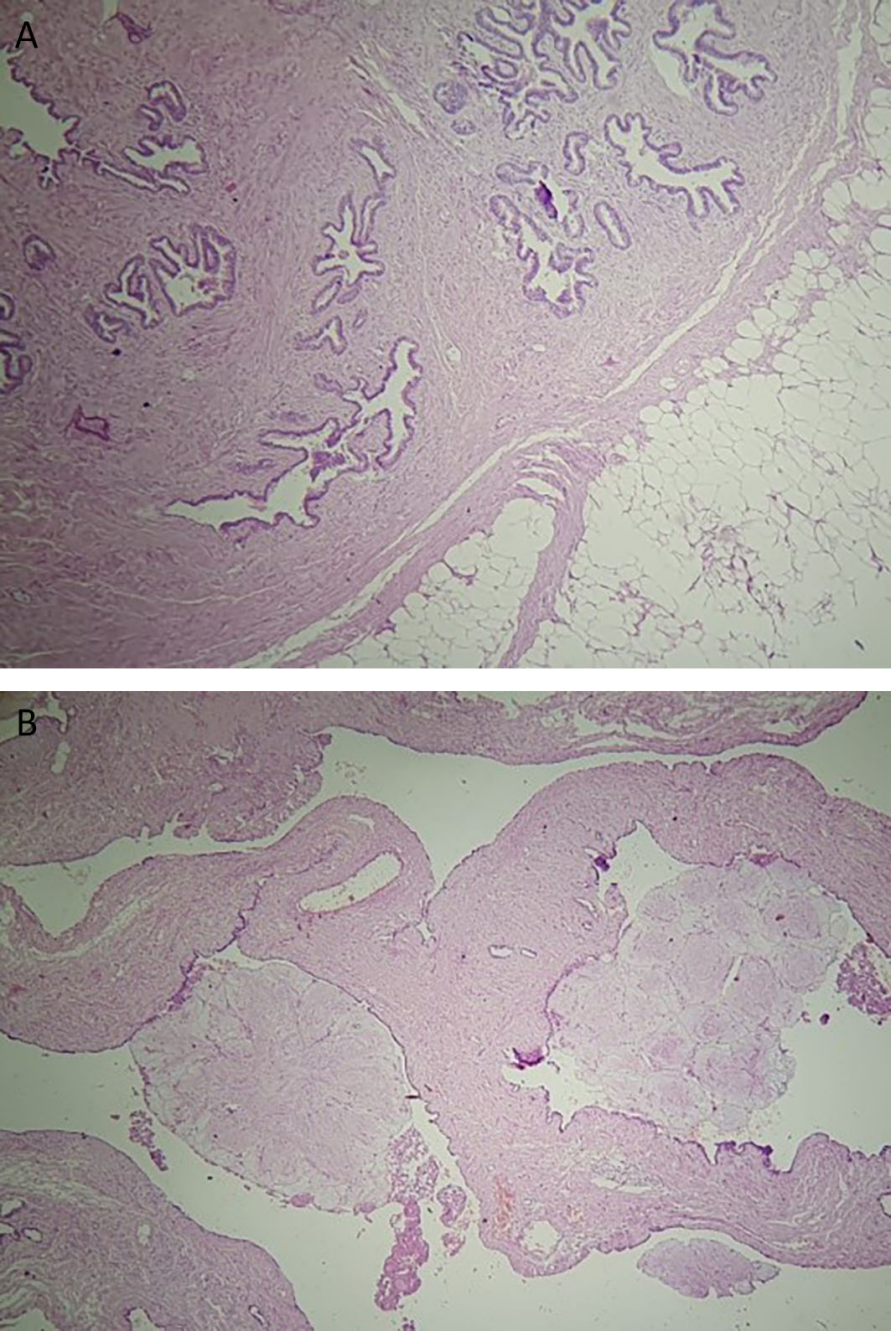
HE*40 Pulmonary hamartoma with adenofibromatous proliferation associated adipose tissue (A) Adenofibromatous pattern with cystic changes and club-like papillae set against a collagenous stroma (B).

The final histology of the tumor showed a well-circumscribed mass measuring 14 × 10 × 5 cm, with predominantly adenofibromatous and lipomatous differentiation, calcifications, and ossified lesions ( Figure 4). There was no evidence of cartilage or muscle tissue. The tumor was covered by a thin fibrous capsule. Additionally, diffuse alveolar hemorrhage lesions were observed in the middle lobe. Follow-up at three weeks post-surgery indicated no adverse outcomes.

## Discussion

Pulmonary hamartomas are benign tumors that often occur in middle-aged adults with male predominance. ^4^ Within the pediatric population, pulmonary hamartomas are significantly rarer. ^5^ It is an incidental finding in most cases, with a diameter ranging from 1 to 8 cm. ^6^ This type of tumor has never been reported as a congenital lesion. Cytogenetic analysis showed abnormalities in chromosomal bands 6p21, 12q14–15, or other regions corresponding to mutations in high-mobility group (HMG) proteins. This group of proteins plays an important role in regulating chromatin architecture and gene expression. ^7^

The pathological pattern of the tumor usually shows predominant chondroid differentiation with a mixture of adipose tissue, fibrous tissue, smooth muscle, and bone, along with entrapped respiratory epithelium **.** Immunohistochemical staining is not necessary for the diagnosis. ^8^ In other cases, the major component can define various subtypes of the tumor: lipomatous, adenoleiomyomatous, and fibrous hamartomas. ^7^^,^^9^ In the current case, the tumor consisted histologically of glandular lumens and fibrous tissue with some calcifications. No evidence of cartilage or muscle tissue damage was observed.

Pulmonary hamartomas are typically asymptomatic. The patient had a medical history of recurrent pneumonia before being referred to our department. Respiratory infections may occur because of mechanical obstruction of the bronchus.

On tomodensitometry, lung hamartoma usually appears as a lobulated nodule with a heterogeneous density and no pleural traction. Characteristic imaging manifestations include the presence of fat (60% of the cases) and a popcorn appearance of calcifications observed in 5-50% of the cases. ^10^ Malignant transformation is exceedingly rare. ^11^

Surgery is indicated for symptomatic masses or those in which malignancy cannot be excluded. ^12^^,^^13^ Enucleation and wedge resection are the most common surgical choices for preserving functional lung tissue. ^14^ However, in our case, tumor resection and middle lobectomy were mandatory because of the large size of the tumor and compression of the surrounding parenchyma.

## Conclusions

Lung hamartomas typically occur in adults and are asymptomatic in most cases; parenchymal resection is rarely required when surgery is indicated. Our case is unusual because of its many peculiarities. A 5-year-old child presented with recurrent pneumonia. Moreover, owing to its large size, the tumor caused parenchymal damage, leading to middle lobectomy during surgery. Finally, the tumor was characterized by predominant adenofibromatous differentiation, with no cartilage. Knowledge of atypical presentations of this neoplasm is crucial to avoid misdiagnosis and to guide appropriate surgical treatment, especially in pediatric patients.

## Consent

Written informed consent was obtained from the patient’s parents for the publication of this case report and accompanying images.

## Data Availability

No data are associated with this article.
