# Supplementary material for: Case Report: Giant lung hamartoma : An usual cause of lobectomy in a five-year child
Source: F1000Res. 2024 Sep 24;13:644. Originally published 2024 Jun 17. [Version 2] doi: 10.12688/f1000research.146993.2 (PMC11881052; doi:10.12688/f1000research.146993.2)
Supplement: Peer review history

# Reviewer report: Reviewer response for version 2

**DOI:** 10.5256/f1000research.171972.r361907

**Published:** 2025-03-04

**Authors:** Sevilay Özmen

**Affiliations:** 1 Department of Pathology, Faculty of Medicine, Atatürk University, Erzurum, Turkey

General Evaluation: This case report reports a rare presentation of a large pulmonary hamartoma in a pediatric patient. The article shows how the rare disease pulmonary hamartoma can present clinically in children. The authors have presented a successful case report with detailed information about the patient's history of recurrent pneumonia, imaging modalities, and surgical intervention. The article also describes such a rare case in the literature and sheds light on the diagnostic and therapeutic processes of similar cases.

 The case highlights the atypical presentations of pulmonary hamartoma and how it may follow a different clinical course in pediatric patients. This may raise awareness in clinical practice.

 It was stated that genetic analysis should be performed in some pulmonary hamartoma cases and some chromosomal abnormalities were observed. However, genetic analysis was not performed for this case and it would be useful to provide more information in this section.

 “HMGA2 fusion in lung hamartomas suggests that these tumors share a similar pathogenesis with other mesenchymal tumors harboring HMGA2-related fusion genes (e.g., chondromas, extraskeletal osteochondromas, lipomas, and leiomyomas)” is stated in reference number 8. However, the source of information regarding molecular genetics is stated as number 7. This information needs to be corrected.

Are enough details provided of any physical examination and diagnostic tests, treatment given and outcomes?

Yes

Is the case presented with sufficient detail to be useful for other practitioners?

Partly

Is sufficient discussion included of the importance of the findings and their relevance to future understanding of disease processes, diagnosis or treatment?

Yes

Is the background of the case’s history and progression described in sufficient detail?

Yes

Reviewer Expertise:

Pulmoner pathology, Hepatopankreato biliary pathology

I confirm that I have read this submission and believe that I have an appropriate level of expertise to confirm that it is of an acceptable scientific standard, however I have significant reservations, as outlined above.

# Reviewer report: Reviewer response for version 2

**DOI:** 10.5256/f1000research.171972.r326621

**Published:** 2024-11-04

**Authors:** Hüseyin Fatih Sezer

**Affiliations:** 1 Department of Thoracic Surgery, Kocaeli University Faculty of Medicine, Kocaeli, Turkey

I reviewed the final version of the article.

Are enough details provided of any physical examination and diagnostic tests, treatment given and outcomes?

Yes

Is the case presented with sufficient detail to be useful for other practitioners?

Partly

Is sufficient discussion included of the importance of the findings and their relevance to future understanding of disease processes, diagnosis or treatment?

Partly

Is the background of the case’s history and progression described in sufficient detail?

Partly

Reviewer Expertise:

The article is a repetition of what is generally known and has no additional features, except for some surgical difficulty.

I confirm that I have read this submission and believe that I have an appropriate level of expertise to confirm that it is of an acceptable scientific standard.

# Reviewer report: Reviewer response for version 1

**DOI:** 10.5256/f1000research.161126.r317823

**Published:** 2024-09-14

**Authors:** Duc T Nguyen, Kenneth J. Nobleza

**Affiliations:** 1 Baylor College Medicine, Houston, Texas, USA

Thank you for presenting this rare pulmonary hamartoma in children. Please find below a couple of comments you may want to consider for improving the manuscript:

 - More details regarding the surgery (pre-, intra-, and post-op) would be needed.

 - As the patient has got recurrent admission for right pneumonia for the past two years, serial results on chest radiography would be helpful.

Are enough details provided of any physical examination and diagnostic tests, treatment given and outcomes?

Yes

Is the case presented with sufficient detail to be useful for other practitioners?

Partly

Is sufficient discussion included of the importance of the findings and their relevance to future understanding of disease processes, diagnosis or treatment?

Partly

Is the background of the case’s history and progression described in sufficient detail?

Partly

Reviewer Expertise:

Pediatrics

We confirm that we have read this submission and believe that we have an appropriate level of expertise to confirm that it is of an acceptable scientific standard, however we have significant reservations, as outlined above.

# Reviewer report: Reviewer response for version 1

**DOI:** 10.5256/f1000research.161126.r294003

**Published:** 2024-06-25

**Authors:** Hüseyin Fatih Sezer

**Affiliations:** 1 Department of Thoracic Surgery, Kocaeli University Faculty of Medicine, Kocaeli, Turkey

Dear Authors

 First of all, I would like to thank you for the work you have done to prepare the article. My criticisms and suggestions about the article are as follows.

 - The purpose of the article should be written more clearly in the introduction, and some more literature information should be added.

 - The words 'case report, children, surgery' should be removed from keywords and more effective words should be added.

 - In the discussion section, pathology is included too much, is the article a surgery article or a pathology article? In addition, information about surgery should be added to the discussion (e.g. post-operative complications, postoperative results, etc.) and why anatomical resection is performed or in which cases it can be performed should be explained more clearly and comprehensively.

 -The conclusion section should be rewritten and unnecessary information should be removed. In addition, the discussion section should include the reasons why the case is considered unusual.

 Unfortunately, the article is a repetition of what is generally known and has no additional features, except for some surgical difficulty.

Are enough details provided of any physical examination and diagnostic tests, treatment given and outcomes?

Yes

Is the case presented with sufficient detail to be useful for other practitioners?

Partly

Is sufficient discussion included of the importance of the findings and their relevance to future understanding of disease processes, diagnosis or treatment?

Partly

Is the background of the case’s history and progression described in sufficient detail?

Partly

Reviewer Expertise:

The article is a repetition of what is generally known and has no additional features, except for some surgical difficulty.

I confirm that I have read this submission and believe that I have an appropriate level of expertise to state that I do not consider it to be of an acceptable scientific standard, for reasons outlined above.
